# δ Subunit‐containing GABA_A_ receptors are preferred targets for the centrally acting analgesic flupirtine

**DOI:** 10.1111/bph.13262

**Published:** 2015-10-18

**Authors:** Felicia Klinger, Mirnes Bajric, Isabella Salzer, Mario M. Dorostkar, Deeba Khan, Daniela D. Pollak, Helmut Kubista, Stefan Boehm, Xaver Koenig

**Affiliations:** ^1^ Department of Neurophysiology and Neuropharmacology, Centre for Physiology and PharmacologyMedical University of ViennaSpitalgasse 23Vienna1090Austria

## Abstract

**Background and Purpose:**

The K_v_7 channel activator flupirtine is a clinical analgesic characterized as ‘selective neuronal potassium channel opener’. Flupirtine was found to exert comparable actions at GABA_A_ receptors and K_v_7 channels in neurons of pain pathways, but not in hippocampus.

**Experimental Approach:**

Expression patterns of GABA_A_ receptors were explored in immunoblots of rat dorsal root ganglia, dorsal horns and hippocampi using antibodies for 10 different subunits. Effects of flupirtine on recombinant and native GABA_A_ receptors were investigated in patch clamp experiments and compared with the actions on K_v_7 channels.

**Key Results:**

Immunoblots pointed towards α2, α3, β3 and γ2 subunits as targets, but in all γ2‐containing receptors the effects of flupirtine were alike: leftward shift of GABA concentration‐response curves and diminished maximal amplitudes. After replacement of γ2S by δ, flupirtine increased maximal amplitudes. Currents through α1β2δ receptors were more enhanced than those through K_v_7 channels. In hippocampal neurons, flupirtine prolonged inhibitory postsynaptic currents, left miniature inhibitory postsynaptic currents (mIPSCs) unaltered and increased bicuculline‐sensitive tonic currents; penicillin abolished mIPSCs, but not tonic currents; concentration‐response curves for GABA‐induced currents were shifted to the left by flupirtine without changes in maximal amplitudes; in the presence of penicillin, maximal amplitudes were increased; GABA‐induced currents in the presence of penicillin were more sensitive towards flupirtine than K^+^ currents. In dorsal horn neurons, currents evoked by the δ‐preferring agonist THIP (gaboxadol) were more sensitive towards flupirtine than K^+^ currents.

**Conclusions and Implications:**

Flupirtine prefers δ‐containing GABA_A_ receptors over γ‐containing ones and over K_v_7 channels.

**Abbreviations**
aEPSC: autaptic EPSCs
aIPSC: autaptic IPSCs
BMI: bicuculline methiodide
CNQX: cyano‐2,3‐dihydroxi‐7‐nitroquinoxaline
DH: dorsal horn
DRG: dorsal root ganglion
mIPSCs: miniature IPSCs
THIP: 4,5,6,7‐tetrahydroisoxazolo(5,4‐c)pyridin‐3‐ol) hydrochloride (= gaboxadol)
TTX: tetrodotoxin

## Tables of Links

**Table:** 

Targets	
**Ligand‐gated ion channels** *^a^*	**Ion channels** *^b^*
GABAA receptor	Kv7 channels
GABAA receptor δ subunit	
GABAA receptor γ2 subunit	


Ligands		
Bicuculline	GABA	Penicillin
CNQX	Insulin	Progesterone
Cytosine	Kynurenic acid	Putrescine
Flupirtine	Midazolam	THIP (gaboxadol)

These Tables list key protein targets and ligands in this article which are hyperlinked to corresponding entries in http://www.guidetopharmacology.org, the common portal for data from the IUPHAR/BPS Guide to PHARMACOLOGY (Pawson *et al*.,^2014^) and are permanently archived in the Concise Guide to PHARMACOLOGY 2013/14 (*^a,b^*Alexander *et al*.,2013a,b).

## Introduction

Flupirtine is a centrally acting analgesic drug that was approved in Europe in 1984 (Miceli *et al*., 2008). Its pain‐relieving activity has been documented in various animal models and in humans (Friedel and Fitton, 1993; Devulder, 2010; Szelenyi, 2013). Although the drug is known not to interact with opioid receptors, its mechanism of action has remained elusive until it was found to activate voltage‐gated K^+^ channels in cultured neurons (Jakob and Krieglstein, 1997). Since then, flupirtine has been marketed as SNEPCO, stands for selective neuronal potassium channel opener (Kornhuber *et a*l., 1999; Szelenyi, 2013). Flupirtine opens native and recombinant K_v_7 channels by shifting the voltage‐dependence to more negative values (Martire *et al*., 2004; Wladyka and Kunze, 2006; Klinger *et al*., 2012), and this was believed to be its sole mechanism of action (Szelenyi, 2013). However, most recently, we have found flupirtine to act simultaneously on K_v_7 channels and GABA_A_ receptors (Klinger *et al*., 2012), which are also known to control nociception (Zeilhofer *et al*., 2009).

These pentameric ligand‐gated ion channels are composed of up to four different types of subunits out of a repertoire of at least 19 proteins (Olsen and Sieghart, 2009). GABA_A_ receptors can be categorized according to their distribution within neurons as synaptic and extrasynaptic receptors respectively. These two receptor groups are also characterized by distinct molecular architectures: synaptic receptors typically contain γ subunits, whereas most extrasynaptic receptors are comprised of δ subunits instead. These different subunit compositions also cause specific pharmacological properties: γ2‐containing receptors are modulated by benzodiazepines, whereas δ‐receptors are highly sensitive towards neurosteroids (Farrant and Nusser, 2005; Brickley and Mody, 2012). Benzodiazepines mediate antihyperalgesic effects by acting at receptors that harbour α2, α3 and/or α5 subunits together with γ2, and agonists that prefer receptors containing these α subunits are being investigated as novel analgesics (Zeilhofer *et al*., 2009). Another GABA_A_ receptor ligand with pain‐relieving properties is THIP (gaboxadol) whose analgesic effects are lost in mice‐lacking GABA_A_ α4 or β3 subunits (Chandra *et al*., 2006; Zeilhofer *et al*., 2009). Most recently, the analgesic action of THIP was reported to involve δ‐containing GABA_A_ receptors (Bonin *et al*., 2011).

Flupirtine was found to increase the potency of GABA in inducing currents through GABA_A_ receptors in central and peripheral neurons without changing maximal current amplitudes. At therapeutic plasma concentrations (<10 μM; Kornhuber *et al*., 1999), the effects of flupirtine on GABA_A_ receptors were more pronounced in dorsal root ganglion (DRG) and spinal dorsal horn (DH) than in hippocampal neurons (Klinger *et al*., 2012).

In the present study, we investigated the expression pattern of GABA_A_ receptor subunits in the aforementioned neuronal tissues. Thereafter, effects of flupirtine were compared for various recombinant as well as native synaptic and extrasynaptic GABA_A_ receptors. The revelation of preferential action at a particular type of GABA_A_ receptors is discussed with reference to the possibility of designing new analgesics.

## Methods

### Membrane preparation and western blot experiments

Rats were killed by decapitation after short CO_2_ asphyxia in accordance with the ARRIVE guidelines and the Austrian animal protection law (http://www.ris.bka.gv.at/Dokumente/BgblAuth/BGBLA_2012_I_114/BGBLA_2012_I_114.pdf) and the Austrian animal experiment by‐laws (http://www.ris.bka.gv.at/Dokumente/BgblAuth/BGBLA_2012_II_522/BGBLA_2012_II_522.pdf) that implement European (directive 2010/63/EU; see http://eur‐lex.europa.eu/LexUriServ/LexUriServ.do?uri=OJ:L:2010:276:0033:0079:en:PDF) in Austrian law. Hippocampi, DRG or DH of the spinal cord was collected from 10–14‐day‐old rats and homogenized in homogenizing buffer (HB) containing (mM): HEPES (10), EDTA (1) and sucrose (300), supplemented with EDTA‐free protease inhibitors (Roche, Vienna, Austria). After centrifugation at 50.000 g, the pellet was resuspended in sucrose‐free HB and subsequently re‐centrifuged at 50.000 g. Pellets were diluted in Laemmli sample buffer to yield concentrations of 1 µg · μL^−1^ protein. Equal amounts (8 µg) of protein were subjected to 10% (SDS‐PAGE). For positive controls, membrane proteins from the whole brain of mice were used for α1, α2, α3, α4, β1, β3 and γ2, from hippocampus for α5 and from cerebellum for β2 and δ subunits. After separation on SDS‐PAGE, proteins were transferred onto nitrocellulose membranes that were blocked in 1.5% milk powder and 0.1% Tween20 in PBS (in mM: NaCl 136.9, KCl 2.7, KH_2_PO_4_ 1.8, Na_2_HPO_4_ 10 and pH 7.4) for 1 h. Membranes were then incubated overnight with one of the following antibodies (1 µg · mL^−1^) directed against GABA_A_ receptor subunits: α1(amino acid residues 328–382); α2(416–424), α3(338–385), α4(379–421), α5(337–388), β1(350–404), β2(351–405), β3(345–408), γ2(1–33) or δ(1–44) (Poltl *et al*., 2003). For visualization, goat anti‐rabbit antibodies linked to alkaline phosphatase [AP‐conjugated goat anti‐rabbit IgG F(abʹ)2, Jackson Immuno Research, West Grove, PA, USA] at a dilution of 1:2000, as well as CDP‐Star® chemiluminescent substrate (Sigma‐Aldrich) at a dilution of 1:1000, were used. Blots were evaluated using a Fluor‐STM MultiImager (Bio‐Rad Laboratories, Hercules, CA, USA) and quantified by the quantity one quantitation software (Bio‐Rad). Two lanes in each blot were also stained for α1 subunits; all images were taken after 10 min. Experiments were performed three times, and each subunit was detected on one blot in duplicates. Relative amounts of GABA_A_ subunits in the various tissues were estimated by comparison with α1 subunits within the same tissue and the same blot. Thereafter, normalized densities were compared for the three tissues investigated.

### Cell cultures and transfections

Primary cultures of rat hippocampal and DH neurons were prepared as described previously (Klinger *et al*., 2012). Micro‐island cultures were obtained as detailed elsewhere (Dorostkar and Boehm, 2007). For heterologous expression of GABA_A_ receptors and K_v_7 channels, tsA 201 cells (a subclone of HEK 293 cells) were cultured in DMEM containing 1 g · L^−1^ glucose and 10% heat‐inactivated fetal calf serum. Cells were transfected using ExGen 500 or TurboFect according to the manufacturer's recommendations, with a transfection ratio of 1:1 for αβ receptors, 1:1:8 for αβγ or αβδ receptors and 1:1 for heteromeric K_v_7.2/K_v_7.3 channels. The day after transfection, cells were seeded at a low density and used for patch‐clamp recordings 24–48 h after transfection. Control experiments were performed in untransfected tsA 201 cells; there, flupirtine failed to enhance any type of current.

### Electrophysiology

Recordings of GABA‐evoked currents and currents through K_v_7 channels were done at room temperature (20–24 °C) using the perforated‐patch method as described previously (Klinger *et al*., 2012). Pipettes were fabricated from borosilicate glass capillaries (GB150‐8P, Science Products, Hofheim, Germany) with a Sutter P97 puller (Sutter Instruments, Novato, CA, USA). Tip resistances were between 2 and 5 MΩ. Pipettes were front filled with internal solution and then back filled with the same solution containing 500 µg · mL^−1^ amphotericin B. Recordings were started after 20–30 min when series resistance had stabilized below 20 MΩ. Recordings of tonic currents were performed at room temperature (20–24 °C) using the whole‐cell patch clamp method to avoid contributions of K_v_7 channels.

The internal solution contained (mM): KCl (140), CaCl_2_ (2), MgCl_2_ (0.7), EGTA (10) and HEPES (10) (pH adjusted to 7.3 with KOH) for measurements of synaptic and GABA‐evoked currents; K_2_SO_4_ (75), KCl (55), MgCl_2_ (8) and HEPES (10), adjusted to pH 7.3 with KOH, for recordings of currents through K_v_7 channels and CsCl (130), TEACl (20), CaCl_2_ (0.24), EGTA (5), glucose (10), HEPES (10) adjusted to pH 7.4 with CsOH (15) for recordings of tonic currents. The external solution consisted of (mM): 140 NaCl, 20 glucose, 10 HEPES, 2.5 CaCl_2_, 2 MgCl_2_, 3 KOH (pH adjusted to 7.4 with NaOH). These solutions result in calculated liquid junction potentials of up to 3.5 mV, which, however, were ignored. Flupirtine was first dissolved in DMSO (30 mM) and then diluted in external solution. DMSO as solvent was used at appropriate concentrations.

Autaptic currents in microculture neurons were evoked by 1 ms depolarizations from −70 to 30 mV. Miniature inhibitory postsynaptic currents (mIPSCs) were recorder in mass cultures in the presence of 0.5 μM tetrodotoxin to block Na^+^ channels and cyano‐2,3‐dihydroxi‐7‐nitroquinoxaline (CNQX, 10 μM) to block mESPCs. Currents through GABA_A_ receptors were elicited by application of agonists (GABA or THIP) to neurons in mass cultures clamped at −70 mV. When a second drug was co‐applied with agonist, cells were exposed to that drug for at least 10 s before and after co‐application. Currents through K_v_7 channels were evoked by ramp hyperpolarizations from −20 to −60 mV for periods of 1 s (Figure 7C). For quantification of the effects of flupirtine, amplitudes measured at a voltage of −30 mV were compared; this corresponds to the voltage at which neuronal K_v_7 channels are approximately half activated (Brown and Passmore, 2009). Cells were continuously superfused, and drugs were applied using a piezo‐switched perfusion fast‐step SF‐77B connected to an eight‐channel perfusion valve control VC‐8 system (Warner Instruments, Hamden, Connecticut, USA). Currents were low‐pass filtered at 2–10 kHz, digitized at 5–20 kHz and stored on an IBM compatible computer (IBM, Armonk, NY, USA). Traces were analysed offline using the Clampfit 10.2 programme (Molecular Devices, Sunnyvale, CA, USA).

### Data analysis, statistics and nomenclature

Values obtained in the presence of solvent (DMSO) or flupirtine were normalized to arithmetic means of reference values obtained before and after the application of solvent and flupirtine respectively. Occasionally, values obtained in the presence of flupirtine were expressed as percentage of values obtained in solvent (% of control). When determining concentration‐response curves for GABA‐evoked currents, 30 μM GABA (in solvent) was applied at the beginning and at the end of recordings to obtain amplitude values then used for normalization. In α1β2γ2S containing receptors, which were investigated first, this was a maximally active concentration (Figure 2A). All GABA concentrations (in solvent as well as in flupirtine, including 30 μM GABA) were applied in between these ‘normalization currents’, and the current amplitudes obtained were divided by the mean of the ‘normalization current’ amplitudes (penicillin was used instead of solvent in Figure 6B). Nonlinear fits of these curves according to Hill equations were obtained using GraphPad Prism (GraphPad Software, San Diego, CA, USA), which also calculates an *F* test on the extra sum of squares to analyse whether fit parameters are shared by two curves.

All data points represent arithmetic means ± SEM; *n* = number of single cells. Statistical analyses of multiple comparisons were obtained by non‐parametric (either Kruskal–Wallis or Friedman test, the latter for paired observations) analyses followed by Dunn's multiple comparison using GraphPad Prism. For comparisons between two groups, non‐parametric Mann–Whitney or Wilcoxon matched pairs tests were employed.

The drug and molecular target nomenclature in this paper conforms to British Journal of Pharmacology's Concise Guide to Pharmacology (Alexander *et al*., 2013a,b).

### Materials

Rat GABA_A_ receptor subunit cDNAs were generously provided by Werner Sieghart, Margot Ernst and Petra Scholze (Center for Brain Research, Vienna, Austria) (Sarto‐Jackson *et al*., 2012) and plasmids for K_v_7.2 and K_v_7.3 channels by Mark Shapiro (San Antonio, TX, USA) (Li *et al*., 2005). Flupirtine, GABA, gaboxadol (4,5,6,7‐tetrahydroisoxazolo(5,4‐c)pyridin‐3‐ol) hydrochloride, midazolam, bicuculline methiodide, kynurenic acid, cyano‐2,3‐dihydroxy‐7‐nitroquinoxaline (CNQX), putrescine, progesterone, poly‐D‐lysine, cytosine, arabinoside, amphotericin B and bulk chemicals were obtained from Sigma‐Aldrich (Vienna, Austria); tetrodotoxin from Latoxan (Rosans, France) and insulin, transferrin and Na‐selenite from Roche (Mannheim, Germany). DMEM, Leibovitz L‐15 medium, penicillin, streptomycin and L‐glutamine were from PAA Laboratories (Pasching, Austria); papain from Worthington (Lakewood, NJ, USA); heat‐inactivated fetal calf serum from Invitrogen (Lofer, Austria); ExGen and TurboFect reagents from Fermentas (St. Leon‐Rot, Germany) and culture dishes from Nunc (Roskilde, Denmark).

## Results

### Differences in GABA_A_ receptor subunit expression in hippocampus, dorsal horn and dorsal root ganglia

**Figure 1:**
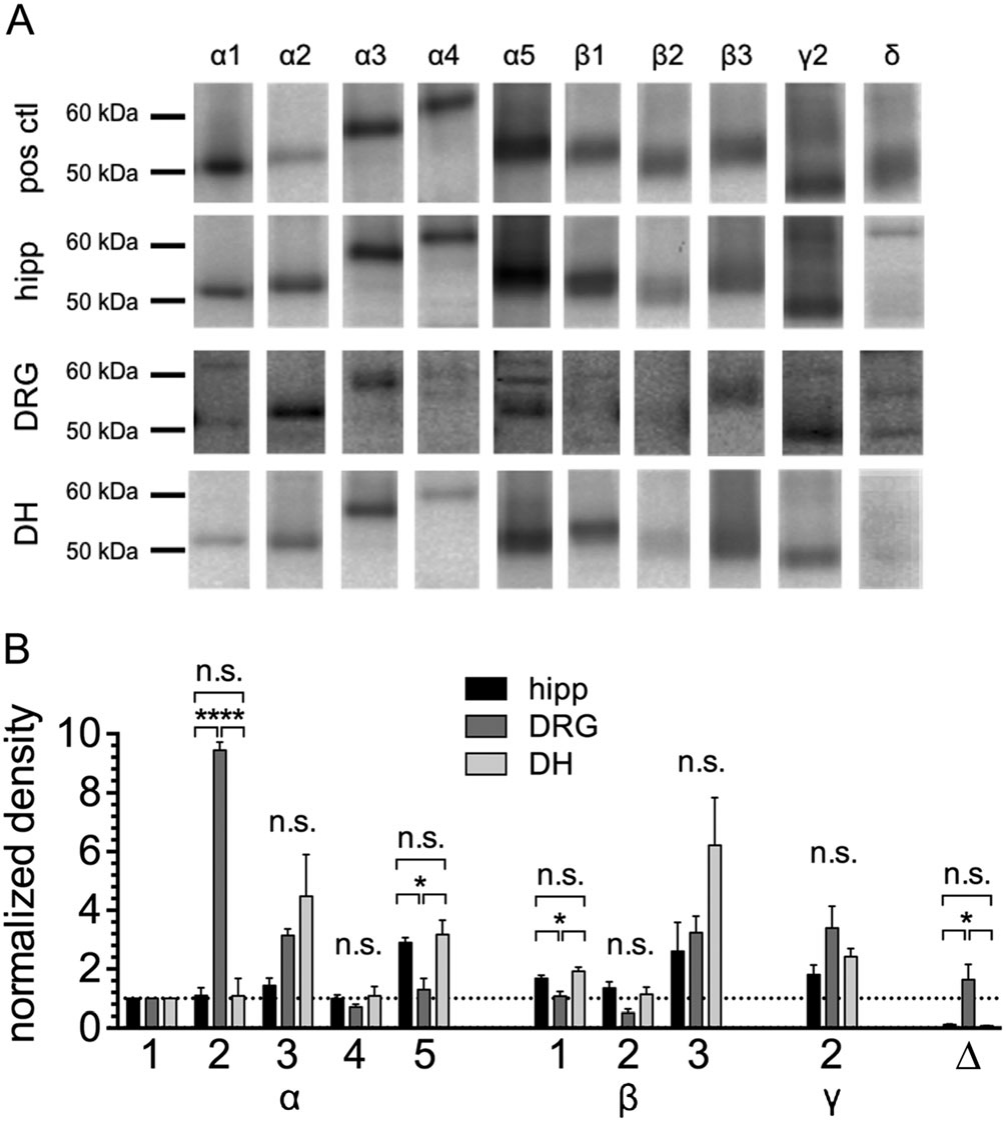
Comparison of the expression pattern of GABA_A_ receptor subunits in hippocampal, dorsal root ganglia and dorsal horn neurons. Hippocampus (hipp), dorsal horn (DH) and dorsal root ganglia (DRG) were collected from 10–14‐day‐old rats, and membrane proteins were prepared. The proteins were separated on 10% polyacrylamide minigels and transferred to membranes, which were then incubated in one of 10 different antibodies directed against various GABA_A_ receptor subunits. As positive controls (pos ctl) showing reference positions and densities of bands, the same amount of membrane protein from whole mouse brain was used for the antibodies against the α1, α2, α3, α4, β1, β3 and γ2 subunits. For the antibody against α5, membrane proteins from mouse hippocampus were used, while mouse cerebellum was used as positive control for the antibodies directed against the β2 and δ subunits. (A) Shows the various bands obtained with the antibodies in membrane preparations of the three different tissues, as observed in one experiment. (B) The densities of all bands were normalized to that of the α1 band within the same tissue obtained in the same experiment; the results show the average values for three independent experiments. * and **** indicate significant differences at *P* < 0.05 and *P* < 0.0001 (ANOVA, followed by Holm–Sidak's multiple comparison *post hoc* test with pooled variance).

To correlate the previously observed differences in the effects of flupirtine on GABA‐evoked currents in DRG, spinal DH and hippocampal neurons (Klinger *et al*., 2012) with certain GABA_A_ receptor subunits, these structures were dissected from 10–14‐day‐old rats, and membrane preparations thereof were subjected to immunoblot analyses. For immunodetection, we used a series of 10 antibodies directed against the following proteins: α1, α2, α3, α4, α5, β1, β2, β3, γ2 and δ subunits of GABA_A_ receptors (Figure 1A).

Individual bands stained by these antibodies exhibited appropriate molecular masses (Figure 1A) as determined previously (Poltl *et al*., 2003). In cases of low abundance of subunits, longer exposure times were required; only then, additional cross‐reactive protein bands became more prominent relative to the subunit and were detected (one additional protein band of 62 kDa labelled by anti‐α_1_ in DRG, one additional protein band of 57 kDa labelled by anti‐α_4_ in DRG, two additional protein bands of 58 and 61 kDa stained by anti‐α_5_ in DRG and DH, one additional protein band of 63 kDa labelled by anti‐γ_2_ in hippocampus and DRG and two additional bands labelled by anti‐δ at 65 and 45 kDa in hippocampus, DRG and DH). As such, additional bands were not observed in all the tissues investigated; they were not investigated any further.

Staining intensities of different antibodies depend on numbers of epitopes recognized, avidities for individual epitopes, interactions with secondary antibodies, time of incubation with alkaline phosphatase substrate and abundance of subunits in the membranes. Therefore, the aforementioned data cannot be used to estimate absolute amounts of GABA_A_ subunits in the tissues investigated. However, in comparison with the positive controls employed for each of these antibodies, it appeared that there was more GABA_A_ receptor subunit expression in hippocampal and DH neurons than in DRG neurons. To compare relative amounts of subunits present in each type of neuronal tissue, staining intensities with each of the antibodies were normalized to that obtained with anti‐α1, as this subunit is widely distributed in the entire nervous system (Olsen and Sieghart, 2009). A respective densitometric analysis (Figure 1B) revealed the following rank orders of predominating subunits: in hippocampus (α)5 > (β)3 > (Γ)2 > (β)1; in DH (β)3 > (α)3 > (α)5 > (Γ)2 and in DRG (α)2 > (Γ)2 > (β)3 > (α)3. With respect to (α)2, (α)5, (β)1 and (Δ), there were significant differences between DRG and the other two tissues (Figure 1B).

### Flupirtine modulates GABA‐evoked currents through recombinant receptors in a subunit‐specific manner

**Figure 2:**
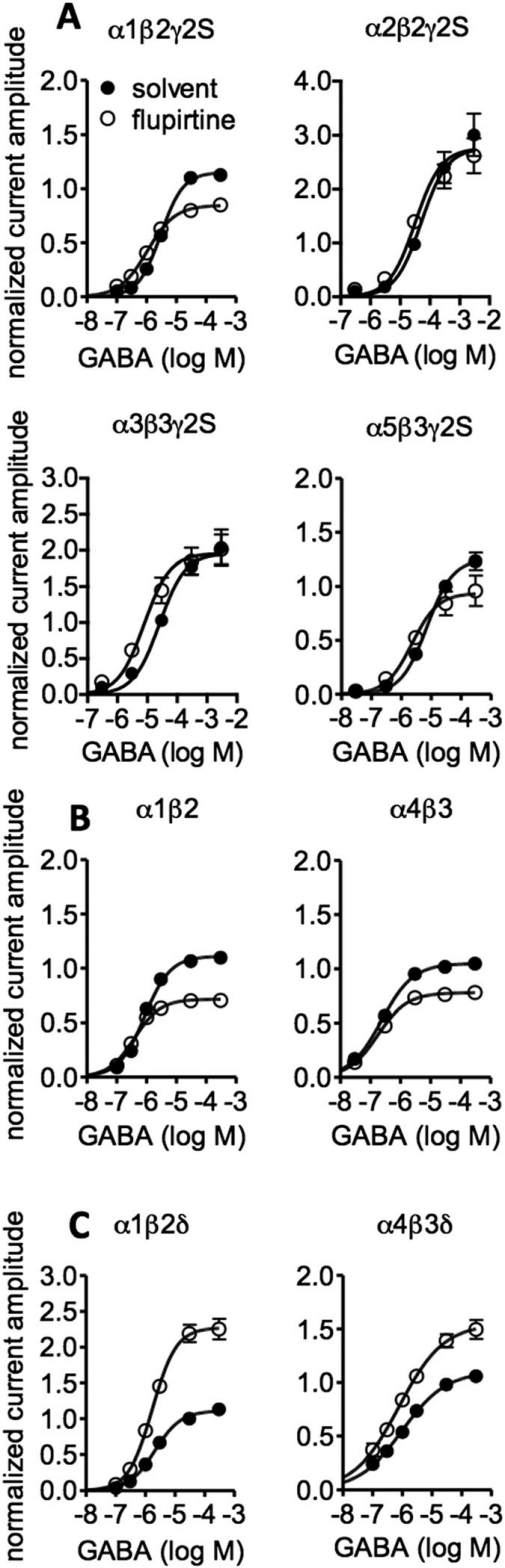
Flupirtine modulates currents through recombinant GABA_A_ receptors. Receptors containing either γ2 (α1β2γ2S, α2β2γ2S, α3β3γ2S and α5β3γ2S; A), δ (α1β2δ and α4β3δ; C) or α and β subunits only (α1β2δ and α4β3; B) were expressed in tsA 201 cells, and currents were evoked by the indicated concentrations of GABA, applied for periods of 3 s, in the continuous presence of either solvent (0.1 % DMSO) or 30 μM flupirtine. Original sample traces are shown in Figure 3A. For the concentration‐response curves, all peak current amplitudes determined in one cell were normalized to the amplitude of the current triggered by 30 μM GABA in the presence of solvent in the very same cell (*n* = 5 to 8). For values of maxima and concentrations for half‐maximal current amplitudes (EC_50_), see Table 1.

As the effects of flupirtine on native GABA_A_ receptors were significantly larger in DRG and DH than in hippocampal neurons (Klinger *et al*., 2012), the above data indicated that GABA_A_ receptors containing α2, α3, β3 and γ2 subunits were the preferred targets for flupirtine. Therefore, the drug was assayed for effects on different GABA_A_ receptor subunit combinations that included the aforementioned proteins (α2, α3, β3 and γ2S); the results obtained were compared with those from receptors typically expressed in the hippocampus (α1β2γ2S and α5β3γ2S; Brickley & Mody, 2012). Initially, concentration‐response curves for GABA‐evoked currents were determined in the presence of a high concentration (30 μM) of flupirtine, as this concentration has previously been shown to affect GABA_A_ receptor currents in all types of neurons tested (Klinger *et al*., 2012). With all these recombinant GABA_A_ receptors, GABA concentrations required for half maximal current amplitudes (EC_50_) were lower in the presence of flupirtine than in its absence (Figure 2A and Table 1). In addition, in two of these receptors (α1β2γ2S and α5β3γ2S), maximal current amplitudes were significantly reduced by flupirtine (Figure 2A and Table 1).

**Table 1:** Hill equation parameters for concentration‐response relationships for GABA‐evoked currents through native and recombinant GABA_A_ receptors in the presence of either solvent or 30 μM flupirtine

	Solvent		Flupirtine (30 μM)	
Receptor	EC_50_ (μM)	*E_max_* (normalized)	EC_50_ (μM)	*E_max_* (normalized)
Hippocampus	9.4 ± 1.0	1.33 ± 0.03	3.5 ± 0.5***	1.25 ± 0.04 n.s.
Hippocampus (penicillin)	1.8 ± 0.2	1.02 ± 0.02	1.2 ± 0.1***	1.46 ± 0.03***
α1β2	0.8 ± 0.1	1.10 ± 0.02	0.3 ± 0.2**	0.71 ± 0.02***
α1β2γ2S	3.0 ± 0.3	1.15 ± 0.02	1.1 ± 0.2***	0.85 ± 0.02***
α1β2δ	2.1 ± 0.2	1.11 ± 0.02	1.7 ± 0.2 n.s.	2.27 ± 0.06***
α2β2γ2S	66.6 ± 18.4	3.03 ± 0.20	24.6 ± 6.4*	2.54 ± 0.14 n.s.
α3β3γ2S	27.0 ± 6.2	2.00 ± 0.10	7.4 ± 2.1**	1.93 ± 0.10 n.s.
α4β3	0.2 ± 0.1	1.05 ± 0.02	0.2 ± 0.01 n.s.	0.78 ± 0.02***
α4β3γ2S	28.0 ± 3.2	1.97 ± 0.07	7.0 ± 1.0***	1.63 ± 0.05***
α4β3δ	1.0 ± 0.02	1.10 ± 0.05	0.8 ± 0.02 n.s.	1.55 ± 0.06***
α5β3γ2S	7.2 ± 1.2	1.25 ± 0.04	2.3 ± 1.0**	0.94 ± 0.06***

Concentration‐response curves of currents through the GABA_A_ receptors listed were obtained in the presence of either solvent or 30 μM flupirtine (*F* test; *n* = 4 − 8; n.s. = no significant difference).

* *P* < 0.05.

** *P* < 0.01.

*** *P* < 0.001 vs. the corresponding values in solvent). Corresponding concentration‐response curves are displayed in Figures 2 and 6.

We next tested for a role of γ2 by expressing receptors lacking this protein (α1β2 and α4β3). In α1β2 receptors, GABA EC_50_ values were reduced in the presence of flupirtine as were maximal current amplitudes. In contrast, EC_50_ values with α4β3 remained unchanged, but maximal effects of GABA were diminished (Figure 2B and Table 1). In receptors containing δ subunits (α1β2δ and α4β3δ), the effect of flupirtine was different: while EC_50_ values remained unaffected, maximal current amplitudes were enhanced (Figure 2C and Table 1). Hence, flupirtine modulated GABA‐evoked currents in a subtype‐specific manner.

### Concentration‐dependence of the effects of flupirtine on recombinant GABA_A_ receptors, comparison with K_v_7 channels

The above results were obtained with flupirtine concentrations above therapeutic plasma levels, which hardly exceed 10 μM (Kornhuber *et al*., 1999). Therefore, three subunit combinations (α1β2γ2S, α1β2δ and α4β3δ) that are known to exist in the CNS (Olsen and Sieghart, 2009) and represent prototypical examples of synaptic (α1β2γ2S) and extrasynaptic (α4β3δ and α1β2δ) receptors, respectively (Brickley and Mody, 2012), were chosen to complete concentration‐response curves for the effects of flupirtine. Currents were evoked by 1 μM (α4β3δ), 2 μM (α1β2δ) and 3 μM (α1β2γ2S) GABA, respectively, which corresponds to the EC_50_ values of the respective receptors (Table 1).

**Figure 3:**
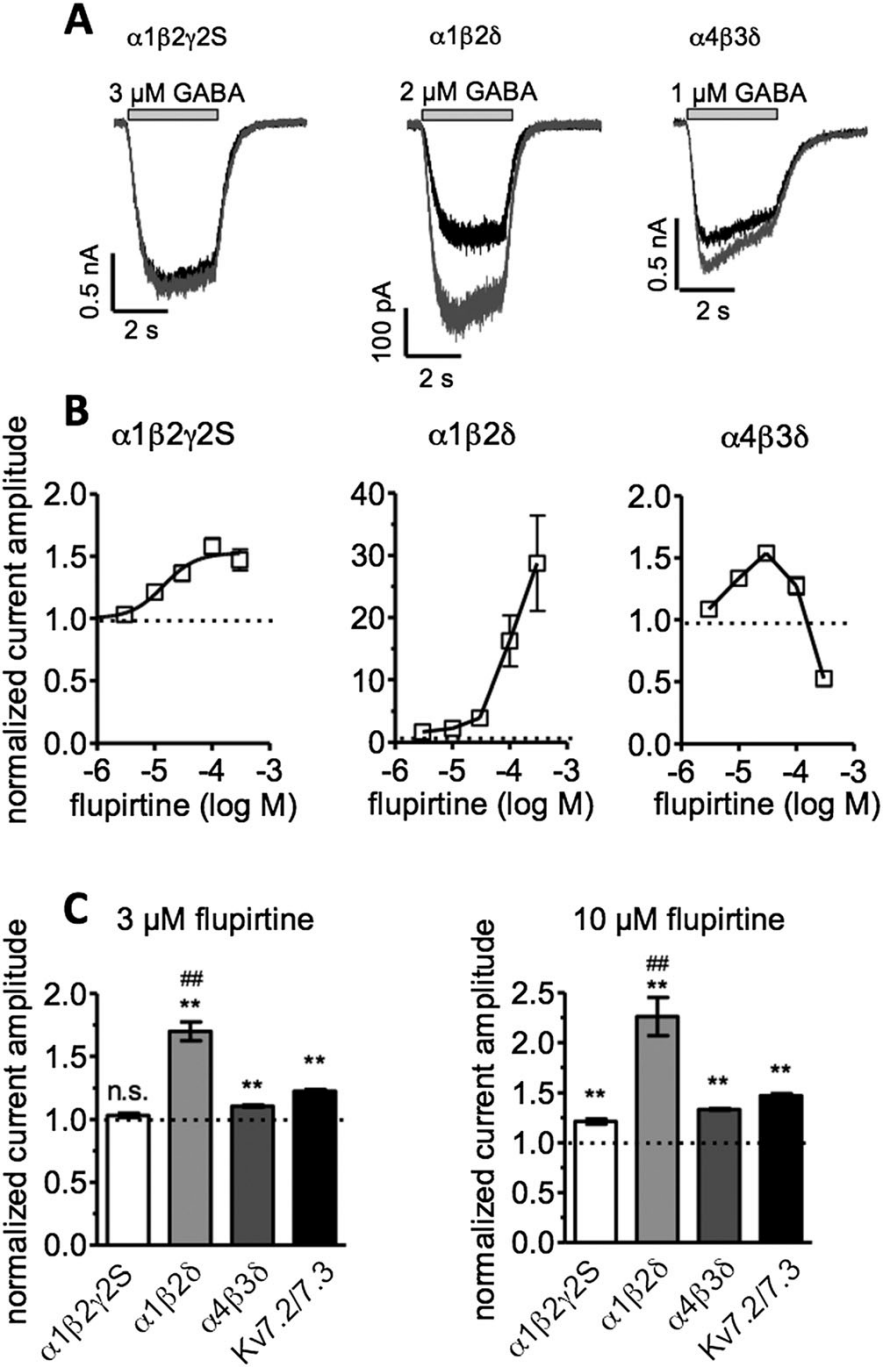
Concentration‐dependence of the effects of flupirtine on recombinant GABA_A_ receptors and comparison with K_v_7 channels. Either GABA_A_ receptors composed of α1β2γ2S, α1β2δ and α4β3δ, respectively, or heteromeric K_v_7.2/7.3 channels were expressed in tsA 201 cells, and currents were evoked either by the application of GABA concentrations corresponding to EC_50_ values (Table 1) or by ramp hyperpolarizations from −20 to −60 mV (for K_v_7 channels). Measurements were performed in the presence of either solvent (<1% DMSO) or the indicated concentrations of flupirtine. Peak amplitudes of GABA‐induced currents and K^+^ current amplitudes at −30 mV were determined respectively. Amplitudes in the presence of the indicated concentrations of flupirtine were normalized to the amplitudes in the presence of solvent. (A) Shows original sample traces of GABA‐evoked currents in presence of solvent (black trace) or 3 μM flupirtine (grey trace). (B) Depicts concentration‐response curves for currents through the indicated receptors (*n* = 5 to 6). Calculated values for α1β2γ2S receptors were 14.3 + 11.0 μM for EC_50_ and 1.53 + 0.05 for maxima. For α1β2δ and α4β3δ receptors, maxima were fixed to the highest values determined (28.8 and 1.5, respectively); then, EC_50_ values were calculated as 108 and 7 μM respectively. (C) A comparison of the effects of 3 and 10 μM flupirtine, respectively, on currents through either GABA_A_ receptors or K_v_7 channels (*n* = 5 to 6); ** indicate significant differences versus solvent at *P* < 0.01; ## indicate significant differences versus all other values at *P* < 0.01.

Flupirtine acted on these receptors in a similar concentration range, but maximal effects on α1β2δ were much larger than those on the other subunit combinations (Figure 3B).

Effects of low concentrations (3 and 10 μM) were compared for these three types of receptors and for recombinant K_v_7.2/7.3 channels. At 3 μM, flupirtine potentiated currents through α1β2δ and α4β3δ, but not those through α1β2γ2S. Currents through K_v_7 channels determined at −30 mV were also enhanced by 3 μM flupirtine. Effects of this concentration on α1β2δ receptors were more pronounced than those on α4β3δ receptors or K_v_7 channels (Figure 3C). In the presence of 10 μM flupirtine, all types of currents were augmented, but again the effects on α1β2δ receptors were more pronounced than those on the other receptors and channels. Hence, flupirtine appeared to influence α1β2δ receptors to the greatest extent, and these are known to be expressed in hippocampal neurons.

### Flupirtine prolongs autaptic inhibitory postsynaptic currents but does not affect mIPSCs, in hippocampal neurons

To investigate the effects of flupirtine on native GABA_A_ receptors, we first chose primary cultures of dissociated hippocampal neurons for the following reasons: (i) such neurons express a wide variety of different GABA_A_ receptor subunits (Sieghart and Sperk, 2002); (ii) in such cultures, phasic and tonic GABAergic inhibition mediated by synaptic and extrasynaptic receptors, respectively, has been found to occur (Yeung *et al*., 2003); (iii) neurons in the hippocampus harbour at least three different types (α1βδ, α4βδ and α5βγ2) of extrasynaptic GABA_A_ receptors (Brickley and Mody, 2012) and finally (iv) IPSCs can be investigated in isolation by using hippocampal neurons in microcultures (Dorostkar and Boehm, 2007).

**Figure 4:**
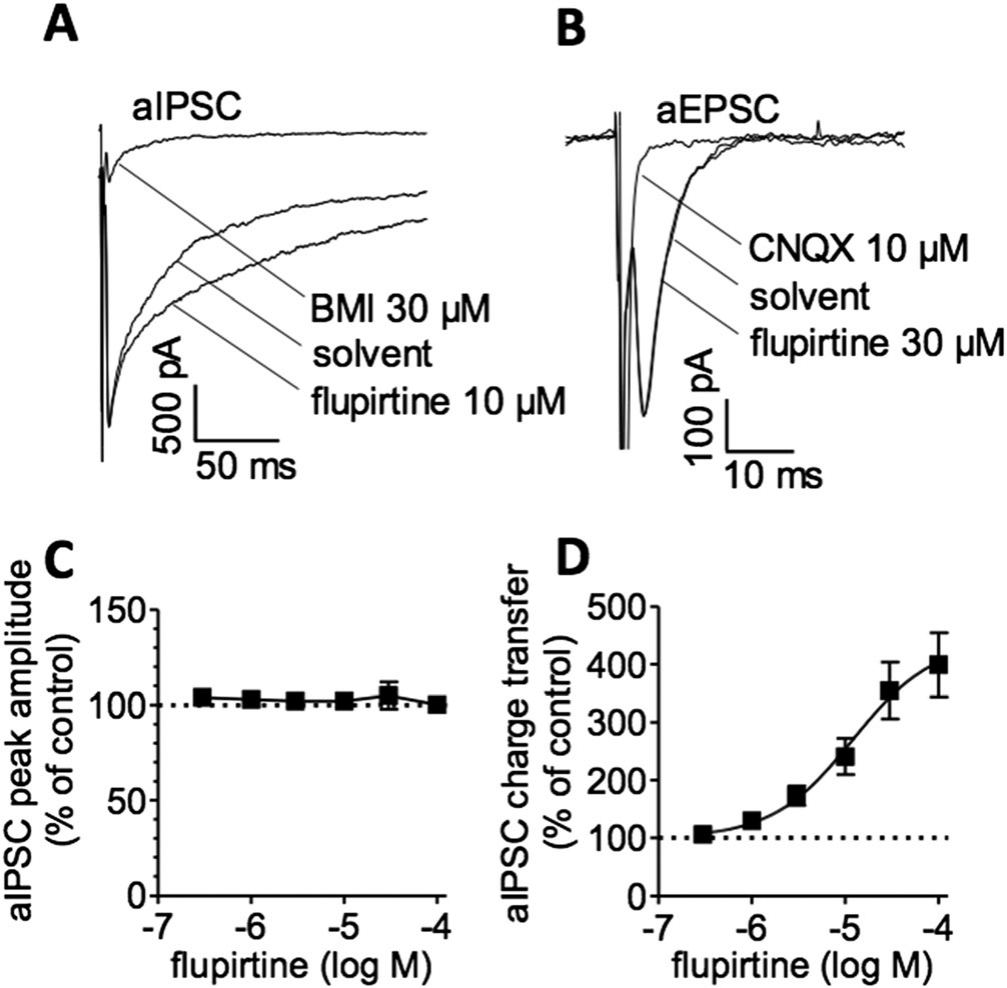
Effects of flupirtine on inhibitory and excitatory autaptic postsynaptic currents in cultured hippocampal neurons. Autaptic inhibitory (aIPSC) or excitatory (aEPSC) postsynaptic currents were elicited in single neuron microcultures by 1 ms depolarizations to +30 mV and recorded at a holding potential of −70 mV in solvent, in the presence of the indicated concentration of flupirtine, and in the presence of 30 μM bicuculline methiodide (BMI) and 10 μM CNQX respectively. (A) and (B) Show representative current traces. (C) Shows the (lack of) effect of flupirtine on peak amplitudes of aIPSCs. (D) Shows the concentration‐response curves for the effect flupirtine on aIPSC charge transfer in the same set of neurons as in (C) (*n* = 6); the EC_50_ value was calculated to be 12 μM.

In such microcultures, autaptic inhibitory postsynaptic currents (aIPSCs) were characterized by prolonged decay times with durations of >100 ms and by a complete block in the presence of bicuculline methiodide (BMI, Figure 4A). Autaptic excitatory postsynaptic currents (aEPSCs), in contrast, were shorter in duration (<<50 ms) and blocked by CNQX (Figure 4B, Boehm and Betz, 1997). When testing for effects of flupirtine on aIPSCs and aEPSCs, respectively, only the former ones were affected (Figure 4). Although flupirtine left aIPSC peak amplitudes unaltered (Figure 4C), the drug caused a prolongation of current decay (Figure 4A). As a consequence, the charge transfer during aIPSCs was increased in a concentration‐dependent manner (Figure 4D). At therapeutic concentrations (3 and 10 μM), charge transfer was significantly increased by 73.4 + 16.9 % (*n* = 6) and 141.2 + 31.3 % (*n* = 6) respectively.

**Figure 5:**
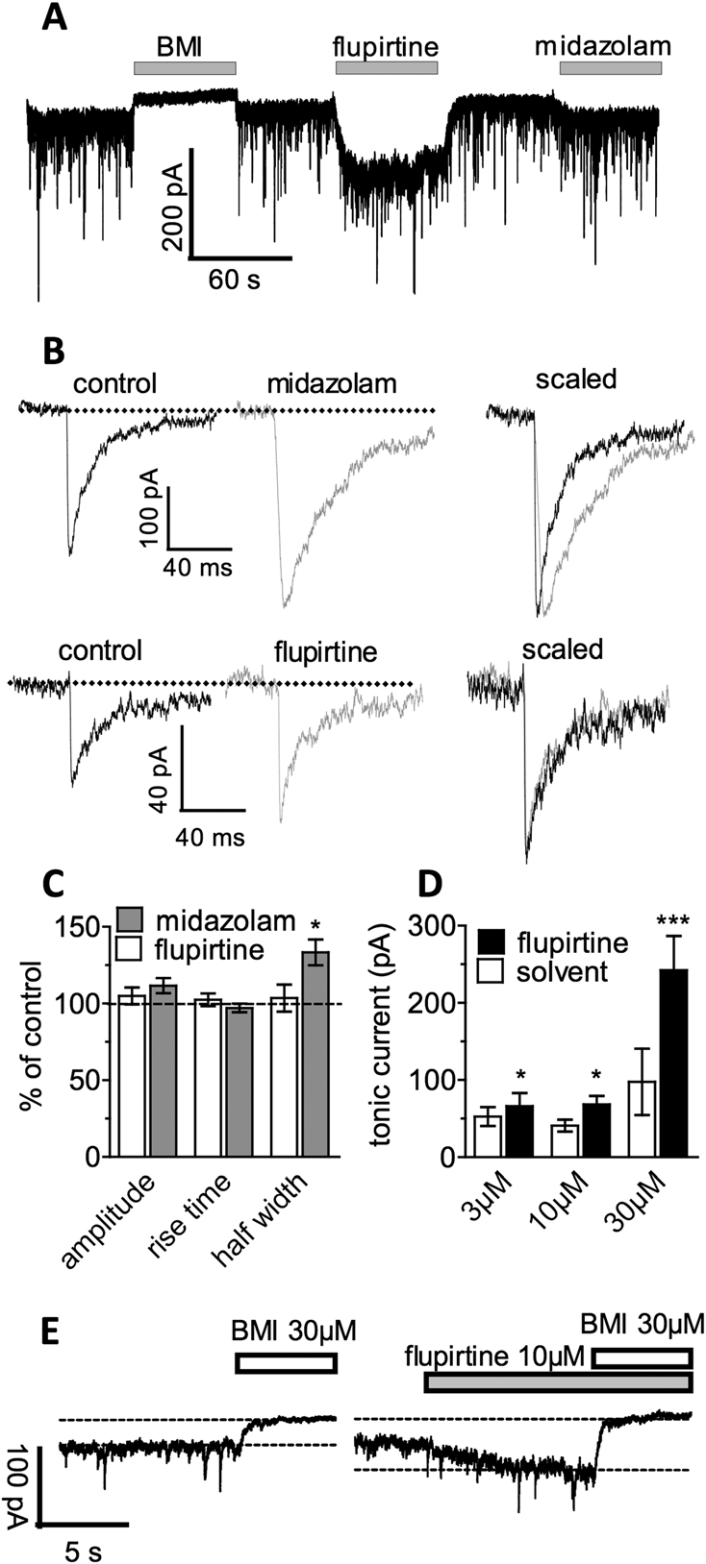
Effects of flupirtine on miniature IPSCs and tonic currents in cultured hippocampal neurons. Spontaneously occurring currents were recorded at a holding potential of −70 mV in 0.5 μM TTX plus 10 μM CNQX (control); 30 μM bicuculline methiodide (BMI), 30 μM flupirtine or 3 μM midazolam were applied as indicated. (A) shows a representative current trace over a period of 6 min; bicuculline methiodide, flupirtine and midazolam were present as indicated by the grey bars. (B) Shows single representative mIPSCs under control conditions and in the presence of flupirtine and midazolam respectively. On the right hand side, mIPSCs were scaled to reach identical maximal amplitudes. (C) Shows mIPSC amplitudes, rise times and half widths in the presence of 30 μM flupirtine or 3 μM midazolam calculated as percentage of the corresponding values obtained in solvent (% of control; *n* = 6; at least 20 events were analysed per neuron); * indicates a significant difference versus solvent at *P* < 0.05. (D) Depicts amplitudes of tonic currents in the presence of 3 (*n* = 11), 10 (*n* = 7) and 30 (*n* = 5) μM flupirtine or solvent. Tonic currents were determined as the difference between baseline currents before and after addition of 30 μM bicuculline methiodide (BMI) in the presence of either solvent or flupirtine. * and *** indicate significant differences versus solvent at *P* < 0.05 and *P* < 0.001 respectively (Wilcoxon matched pairs test). (E) Shows representative traces of tonic currents in one neuron; bicuculline methiodide (BMI) and flupirtine were present as indicated by the bars.

For comparison with evoked autaptic currents, mIPSCs were measured. In the presence of 0.5 μM tetrodotoxin and 10 μM CNQX, such mIPSCs occurred at a frequency of approximately 1 Hz (Dorostkar and Boehm, 2007). Mean amplitudes of mIPSCs amounted to 84 + 16 pA, mean rise times were 1.4 + 0.2 ms and mean widths of events at the level of half of the amplitude (half width) was 13.8 + 1.7 ms (*n* = 6; Figure 5A). In the presence of bicuculline, no similar events were observed. This GABA antagonist not only prevented mIPSCs but also shifted the holding current to more positive values (Figure 5A). In the presence of 30 μM flupirtine, all characteristics of mIPSCs remained unaltered (Figure 5B and C). Holding currents, however, were shifted towards more negative values (Figure 5A and D). For comparison, the benzodiazepine midazolam (3 μM) increased half widths of mIPSCs without altering amplitudes or rise times (Figure 5A, B and C). In addition, midazolam caused a slight shift of holding currents in an inward direction (Figure 5A). The concentration dependence of the effects of flupirtine on tonically active GABA_A_ receptors was quantified by adding bicuculline methiodide to block tonic GABAergic currents. Amplitudes of tonic currents that were block by bicuculline (difference in current amplitudes before and after the addition of 30 μM bicuculline) were enhanced by 3 to 30 μM flupirtine in a concentration‐dependent manner.

### Flupirtine differentially modulates currents through GABA_A_ receptors involved in phasic and tonic inhibition respectively

**Figure 6:**
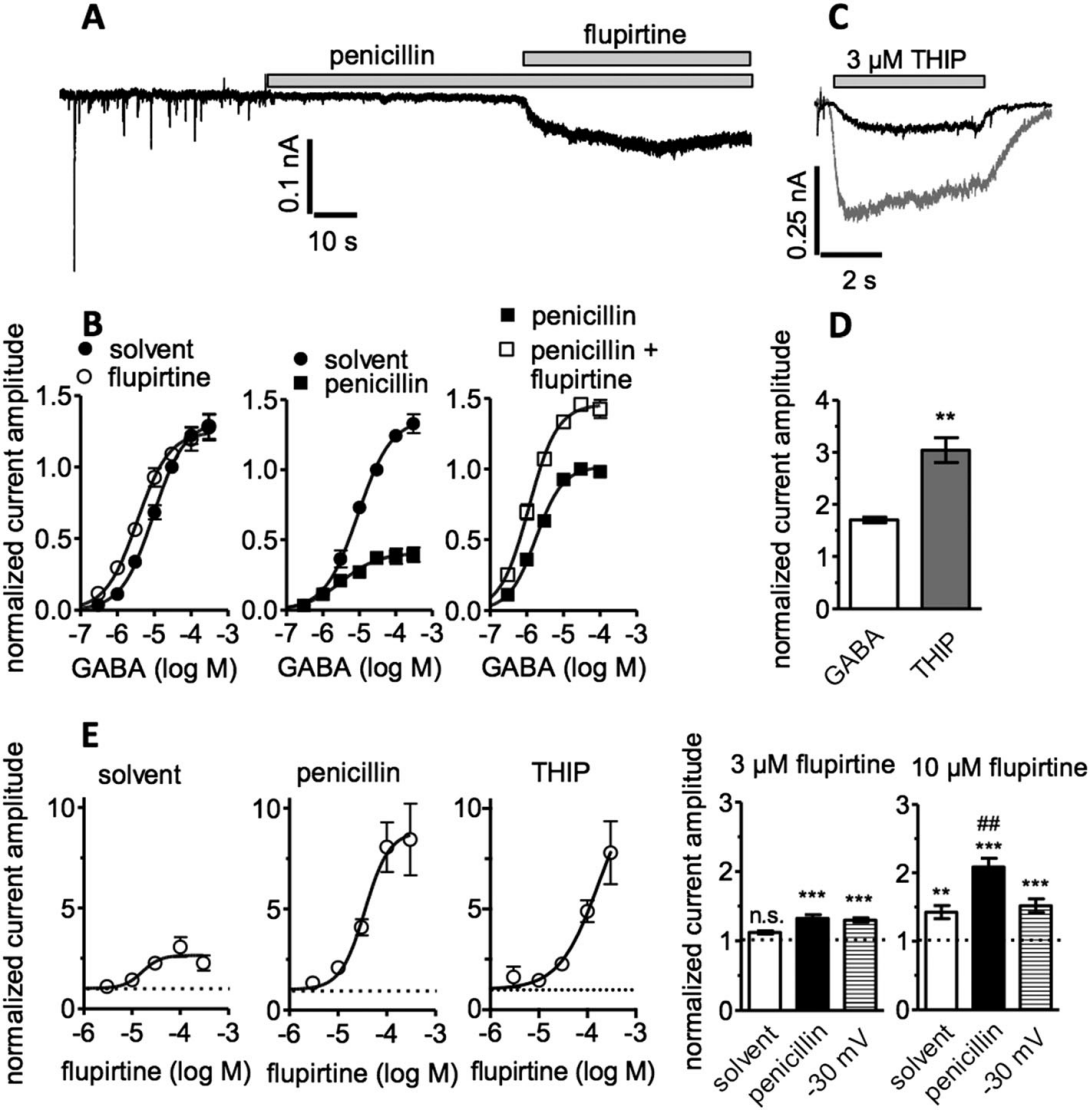
Flupirtine modulates GABA‐ and THIP‐evoked currents in hippocampal neurons. GABA‐ and THIP‐induced currents were measured at a holding potential of −70 mV; K^+^ currents were evoked by ramp hyperpolarizations from −20 to −60 mV. (A) Shows an original trace of spontaneous currents at −70 mV; 5 mM penicillin and 30 μM flupirtine were present as indicated by the bars. (B) Shows concentration‐response curves for currents evoked by the indicated concentrations of GABA in the presence of solvent (0.1% DMSO), 30 μM flupirtine, 5 mM penicillin or 30 μM flupirtine plus 5 mM penicillin. For each cell, current amplitudes were normalized to that evoked by 30 μM GABA in the presence of solvent and penicillin plus solvent respectively (*n* = 4 to 6). For values of maxima and concentrations for half‐maximal currents (EC_50_), see Table 1. (C) Shows an original trace; currents were evoked by 3 μM THIP as indicated by the bar in the presence of either solvent (black trace) or 30 μM flupirtine (grey trace). (D) Compares the effects of 30 μM flupirtine on currents evoked either by 3 μM GABA in the presence of 5 mM penicillin (GABA; *n* = 4) or by 3 μM THIP (*n* = 6). Current amplitudes in the presence of flupirtine were normalized to those in the presence of solvent. (E) Concentration‐response curves for the effects of flupirtine on currents evoked either by 3 μM GABA (solvent; *n* = 6), 1 μM GABA in the presence of 5 mM penicillin (penicillin; *n* = 10) and 3 μM THIP (*n* = 5) respectively. Current amplitudes in the presence of flupirtine were normalized to those obtained in the presence of solvent (THIP) and penicillin plus solvent (penicillin) respectively. (F) A comparison of the effects of 3 and 10 μM flupirtine, respectively, either on GABA‐evoked currents in the absence (solvent) and presence of penicillin or on K^+^ current amplitudes at –30 mV (*n* = 6 to 10); ** and*** indicate significant differences versus solvent at *P* < 0.01 and *P* < 0.001, respectively; ## indicate significant differences versus all other values at *P* < 0.01.

Phasic and tonic GABAergic currents of hippocampal neurons, whether in cultures of dissociated neurons or in brain slices, display distinct pharmacological and biophysical properties (Bai *et al*., 2001). In particular, they can be separated by penicillin, which selectively blocks synaptic currents (Yeung *et al*., 2003). Penicillin (5 mM) did not affect holding currents but abolished mIPSCs (Figure 6A). Nevertheless, in the presence of penicillin, flupirtine was still able to induce downward deflections in holding currents, and apparent inward currents triggered by 30 μM flupirtine (96.8 + 22.4 pA) were not altered by penicillin (85.0 + 21.2 pA; *n* = 3).

To confirm the above results on currents induced by endogenously released GABA, currents evoked by the exogenous application of GABA were determined in the presence of either flupirtine or solvent. The concentration‐response curve for such GABA‐evoked currents was shifted to the left by 30 μM flupirtine without any change in maximal current amplitudes (Figure 6B and Table 1). Penicillin (5 mM), in contrast, significantly reduced maximal current amplitudes and decreased the concentration required to trigger currents with half maximal amplitudes (Table 1 and Figure 6B). In the presence of this penicillin concentration, 30 μM flupirtine not only caused a small leftward shift in the concentration‐response curve but also enhanced maximal current amplitudes (Figure 6B and Table 1). Thus, when receptors involved in phasic GABAergic inhibition were blocked, the action of flupirtine on GABA_A_ receptors was drastically altered.

To confirm that this change in the effect of flupirtine was not due to a modulatory effect of penicillin, 3 μM THIP (gaboxadol) was used to induce currents through GABA_A_ receptors (Figure 6C). At such low concentrations, THIP activates predominantly, if not exclusively, δ‐containing receptors (Maguire *et al*., 2005). Currents induced by THIP were augmented by 30 μM flupirtine to a greater extent than currents induced by 3 μM GABA in the presence of 5 mM penicillin (Figure 6D). Thus, flupirtine mainly enhanced currents through hippocampal GABA_A_ receptors that were insensitive towards penicillin, but sensitive towards THIP.

### Concentration‐dependence of the effects of flupirtine on GABA_A_ receptors in hippocampal neurons, comparison with K_v_7 channels

Concentration‐response curves for the effects of flupirtine on GABA_A_ receptors were collected in the presence and absence of 5 mM penicillin. Currents were evoked by 1 μM (penicillin) or 3 μM (control) GABA, the concentrations previously used for α4β3δ and α1β2γ2S receptors (Figure 3). These concentrations correlate to about one third of the EC_50_ values for GABA in hippocampal neurons in the presence or absence of penicillin (Table 1). Flupirtine caused a maximal increase in current amplitudes by a factor of almost 9 in the presence of penicillin, but only by a factor of 2.5 in the presence of solvent. The effects of flupirtine were half maximal at 16.2 + 5.4 μM and 35.4 + 11.4 μM in the presence and absence of penicillin respectively (Figure 6E). When considering these differences, one has to bear in mind that absolute amplitudes of GABA‐evoked currents in the presence of penicillin were significantly smaller than those in its absence (Figure 6B, middle panel).

To provide additional evidence that the huge effects of flupirtine observed in the presence of penicillin were not due to the presence of the antibiotic, currents were also evoked by 3 μM THIP, which is selective for δ‐containing receptors (Maguire *et al*., 2005). With THIP as agonist, flupirtine increased maximal current amplitudes by a factor of 8 (Figure 6E). This shows that flupirtine acts more effectively on penicillin‐insensitive and THIP‐sensitive than on the entire population of GABA_A_ receptors in hippocampal neurons.

Effects of low flupirtine concentrations on GABA_A_ receptors were compared with their effects on currents evoked through K^+^ channels determined at −30 mV. At a concentration of 3 μM, flupirtine enhanced GABA‐evoked currents in the presence of penicillin (but not those in its absence) as well as the K^+^ currents (Figure 6F). In the presence of 10 μM flupirtine, both types of GABA currents as well as the K^+^ currents were increased; the effect on GABA‐evoked currents in the presence of penicillin was more pronounced than those on the other currents (Figure 6F).

### Concentration‐dependence of the effects of flupirtine on δ‐containing GABA_A_ receptors in DH neurons, comparison with K_v_7 channels

**Figure 7:**
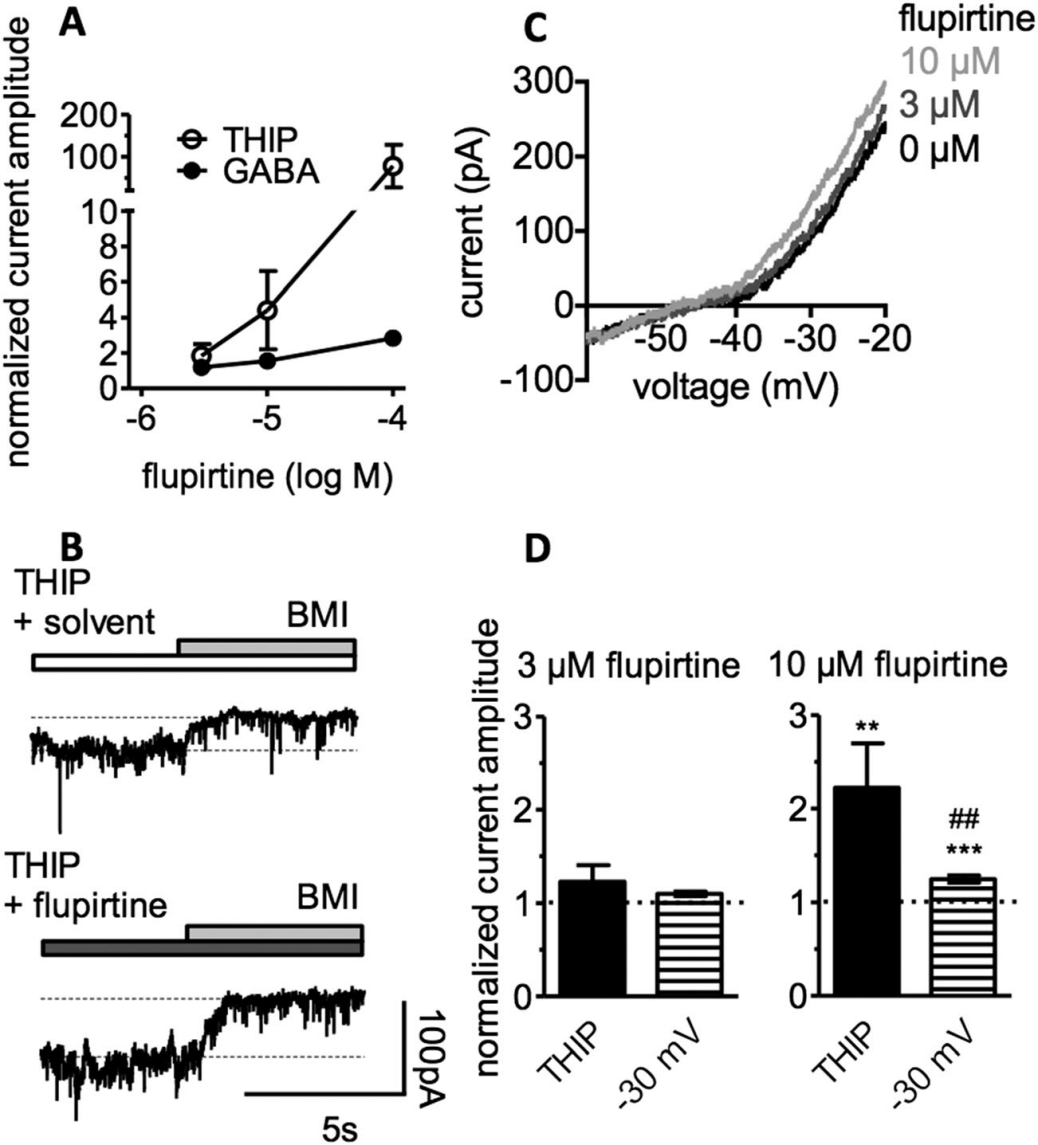
Flupirtine modulates GABA‐ and THIP‐evoked currents in dorsal horn neurons. Currents were induced by 3 μM THIP or 3 μM GABA at a holding potential of −70 mV in the presence of solvent or flupirtine. (A) Concentration‐response curves for the effects of flupirtine on currents evoked either by THIP or GABA. Current amplitudes in the presence of flupirtine were normalized to those obtained in the presence of solvent (0.03 to 0.3 % DMSO; *n* = 4 to 5). (B) Original traces of spontaneous currents at −70 mV; 3 μM THIP in either solvent (0.03 % DMSO) or 10 μM flupirtine as well as 30 μM bicuculline methiodide (BMI) were present as indicated by the bars. (C) Currents evoked by ramp hyperpolarizations from −20 to −60 mV in the presence of solvent (0 μM flupirtine; 0.03 % DMSO) or 3 and 10 μM flupirtine. (D) A comparison of the effects of 3 and 10 μM flupirtine on THIP‐evoked currents (THIP) and outward currents at −30 mV, respectively (*n* = 8); ** and*** indicate significant differences versus solvent (0.01 % and 0.03 % DMSO, respectively) at *P* < 0.01 and *P* < 0.001, respectively; ## indicate significant differences versus currents evoked by THIP at *P* < 0.01.

In DH neurons, flupirtine had been found to enhance GABA‐evoked currents to the same extent as K^+^ currents measured at −30 mV (Klinger *et al*., 2012). To elicit currents primarily through δ‐containing GABA_A_ receptors, THIP (gaboxadol) was used at the same concentration as in hippocampal neurons (3 μM) and triggered small currents (12.5 + 2.4 pA; *n* = 8). For comparison, currents evoked by 3 μM GABA were more than 10‐fold larger (226.7 + 71.0 pA; *n* = 4). Flupirtine enhanced both, THIP‐ and GABA‐induced currents in a concentration‐dependent manner, but the effects on the currents elicited by THIP were more pronounced (Figure 7A). THIP‐evoked currents in the presence of either solvent or 3 and 10 μM flupirtine, respectively, were further quantified by adding bicuculline methiodide to block THIP‐evoked and spontaneous tonic GABAergic currents; the difference between current amplitudes before and after the addition of this GABA antagonist in the presence of flupirtine was normalized to the same difference in the presence of solvent (Figure 7B and D). For comparison, voltage ramps from −20 to −60 mV were applied to elicit K^+^ currents. Such currents were also enhanced by 3 and 10 μM flupirtine (Figure 7C). This increase was again quantified at a voltage of −30 mV (Figure 7D). By direct comparison, enhancement of THIP‐induced currents by 10 μM flupirtine was siginificantly larger than that of K^+^ currents (Figure 7D).

## Discussion

Here, we identified for the first time δ‐containing GABA_A_ receptors as prime targets for flupirtine, a veteran analgesic drug. Flupirtine is marketed as SNEPCO and generally believed to achieve its therapeutic effects through an action on K_v_7 channels (Szelenyi, 2013). Recently, native GABA_A_ receptors and K_v_7 channels of neurons in pain pathways were found to be facilitated by flupirtine to the same extent, whereas hippocampal GABA_A_ receptors turned out to be insensitive to flupirtine (Klinger *et al*., 2012). As shown here, DRG and DH neurons express much higher relative levels of GABA_A_ α2 and α3 subunits than hippocampal neurons. Previous immunohistochemical investigations indicated that α2 and α5 subunits prevail in the hippocampus (Yu *et al*., 2006) while α3 predominates in the spinal cord (Bohlhalter *et al*., 1996). In DRG neurons, mRNA for α2 has been found to dominate (Ma *et al*., 1993). However, when testing for an α‐subunit specificity of flupirtine using recombinant receptors (α1, α2, α3, α4 or α5 expressed together with β2 or β3 and γ2S), effects on EC_50_ values for GABA were similar as they were reduced by factors of 2.7 (α1β2γ2) to 4 (α4β3γ2). αβ GABA_A_ receptors were modulated by flupirtine in more or less the same way as γ‐containing ones. In most of these receptors (with the exception of α2β2γ2 and α3β3γ2), flupirtine reduced maximal current amplitudes by 18 to 35% and hence displayed properties of a non‐competitive antagonist. In contrast, when δ subunits were co‐expressed with α and β, flupirtine acted as a positive allosteric modulator and markedly enhanced maximal current amplitudes. At flupirtine concentrations of <10 μM, γ‐containing receptors were less sensitive than δ‐containing ones; moreover, the enhancement of currents through α1β2δ receptors was even more pronounced than the increase in currents through K_v_7.2/K_v_7.3 channels.

In hippocampal neurons, several subunit compositions have been reported for synaptic and extrasynaptic GABA_A_ receptors: amongst synaptic receptors, the combination α1β2γ2 is found most frequently, whereas α1βδ, α4βδ and α5βγ‐containing pentamers constitute extrasynaptic receptors (Farrant and Nusser, 2005; Olsen and Sieghart, 2009; Brickley and Mody, 2012). Flupirtine increased charge transfer during aIPSCs in hippocampal neurons in microcultures without affecting peak currents; mIPSCs, in contrast, remained unaffected. At first sight, this appears contradictory. However, IPSCs are known to comprise two components: a rapidly and a slowly decaying one. The fast component is prolonged by benzodiazepines that do not affect the slow component (Puia *et al*., 1994; Roepstorff and Lambert, 1994). Conversely, the GABA uptake inhibitor tiagabine did not alter the rapid, but only the slow component (Roepstorff and Lambert, 1994). Similarly, SKF‐89976A, another GABA uptake inhibitor, had no effect on the initial decay phase but prolonged the late phase of evoked IPSCs in CA1 hippocampal neurons. In these neurons, spontaneous IPSCs or IPSCs evoked by weak stimuli were not affected by SKF‐89976A, which was explained by insufficient GABA spillover from the synaptic cleft into the regions containing extrasynaptic GABA_A_ receptors (Isaacson *et al*., 1993). In accordance with these results, IPSCs have been shown to decay significantly slower in hippocampal neurons of wild‐type mice than in such neurons from mice‐lacking GABA_A_ receptor δ subunits that are localized extrasynaptically (Wei *et al*., 2003; Mangan *et al*., 2005). Moreover, NO711, another specific GABA uptake inhibitor failed to affect IPSCs in granule neurons from δ^‐/‐^ mice but significantly prolonged the slow decay time of evoked IPSCs in wild‐type neurons (Wei *et al*., 2003). Taken together, late phases of IPSCs involve spillover of GABA onto extrasynaptic receptors containing δ subunits, and flupirtine acted on these slow components. In contrast, mIPSCs were not affected by flupirtine but were sensitive towards a benzodiazepine, as are the fast components of evoked IPSCs (Puia *et al*., 1994; Roepstorff and Lambert, 1994).

The above considerations indicate that flupirtine acted preferentially on extrasynaptic GABA_A_ receptors. This was confirmed by the finding that flupirtine, while leaving mIPSCs unaltered, enhanced baseline currents, which were blocked by bicuculline. Extrasynaptic receptors mediate tonic GABAergic conductances, whereas phasic GABAergic inhibition relies on synaptic GABA_A_ receptors (Farrant and Nusser, 2005; Brickley and Mody, 2012). However, GABA spillover due to synaptic activity may lead to phasic activation of extrasynaptic receptors, even though the population of extrasynaptic receptors mediating this type of phasic inhibition is not identical to that mediating tonic inhibition (Bright *et al*., 2011). In cultures of hippocampal neurons, tonic GABAergic inhibition can be separated from phasic inhibition by penicillin, which selectively blocks the latter (Yeung *et al*., 2003). Accordingly, mIPSCs were abolished by 5 mM penicillin, whereas baseline currents and their enhancement by flupirtine remained unaltered. In parallel, penicillin drastically altered the effects of flupirtine on GABA‐evoked currents in hippocampal neurons thereby confirming that flupirtine exerted differential effects on synaptic and extrasynaptic GABA_A_ receptors.

Even though penicillin causes less inhibition in δ than in γ2‐containing receptors, the preferential block of phasic inhibition is also related to the conditions of receptor activation: currents evoked by high GABA concentrations are reduced to a greater extent than currents elicited by lower concentrations, and peak current amplitudes are efficiently diminished, whereas steady‐state current levels are hardly affected (Feng *et al*., 2009). Thus, the switch in the effects of flupirtine on hippocampal GABA_A_ receptors in the presence of penicillin was likely due to different sensitivities of γ2‐containing and δ‐containing heteromers. This was confirmed by using 3 μM THIP as the agonist, at a concentration that prefers δ‐containing receptors and does not activate synaptic GABA_A_ receptors (Maguire *et al*., 2005).

Concentration‐response curves for the action of flupirtine on GABA‐evoked currents in hippocampal neurons revealed a maximal enhancement by a factor of <3 in the absence of penicillin and by a factor of >8 in its presence. Currents evoked by 3 μM THIP were also enhanced up to eightfold. Moreover, at 3 μM flupirtine, only GABA‐evoked currents in the presence of penicillin were increased. At 10 μM flupirtine, GABA‐evoked currents of hippocampal neurons in the presence of penicillin were augmented more than K^+^ currents. Likewise, in DH neurons, THIP‐induced currents were enhanced by 10 μM flupirtine significantly more than K^+^ currents. Previously, the population of GABA_A_ receptors in cultured DRG and DH neurons had been found to be more sensitive towards flupirtine than the population in cultured hippocampal neurons (Klinger *et al*., 2012). The present results reveal a preference of flupirtine for δ‐containing receptors. In the case of DRG, this corresponds with a comparably high level of δ subunit expression. In the case of DH neurons, the correlation with the expression levels of GABA_A_ receptor subunits is less conceivable. Nevertheless, the data obtained with THIP also confirm the importance of δ‐containing receptors in DH neurons (see also Bonin *et al*., 2011).

With respect to the present results, one needs to consider levels of therapeutic flupirtine concentrations in the central nervous system. Flupirtine is administered in oral doses of up to 200 mg as often as four times a day; given a plasma half‐life of about 10 h, four regularly spaced doses a day may lead to more than twofold accumulation (Friedel and Fitton, 1993). A single dose of 200 mg results in plasma concentrations of around 5 μM (Hummel *et al*., 1991), 80 % of which are bound by plasma proteins (Friedel and Fitton, 1993). Thus, free plasma concentrations after single doses are in the range of 1 to 2 μM. EC_50_ values for the facilitation of K_v_7 channels are 5 μM (Klinger *et al*., 2012). Hence, such free plasma concentrations can hardly affect extrasynaptic GABA_A_ receptors or K_v_7 channels. More relevant than free plasma concentrations are concentrations in the central nervous system. Even though flupirtine is reported to be evenly distributed throughout the entire organism, direct measurements of brain concentrations have not been reported (Friedel and Fitton, 1993). However, a close structural analogue, retigabine, has been found to achieve sixfold higher concentrations in the brain in comparison with plasma (Sotty *et al*., 2009). Thus, one may surmise a similar brain accumulation of flupirtine and consequently expect concentrations of up to 10 μM flupirtine in the central nervous system. In any case, at low micromolar concentrations, flupirtine's action on δ‐containing GABA_A_ receptors is at least as pronounced as (if not even larger than) its action on K_v_7 channels. Therefore, both families of proteins must be viewed as commensurate targets for this analgesic.

In summary, the present results reveal extrasynaptic δ‐containing rather than synaptic γ2‐containing GABA_A_ receptors as targets for flupirtine. Such δ‐containing receptors are known to mediate antinociceptive effects (Bonin *et al*., 2011) and turn out to be at least as sensitive as K_v_7 channels towards therapeutic flupirtine concentrations. Hence, flupirtine should be regarded an analgesic with a dual mechanism of action: the opening of δ‐containing GABA_A_ receptors and of K_v_7 channels. We therefore suggest that flupirtine should be characterized as a GABA and potassium channel opener rather than SNEPCO. As such, it may serve as a template for the development of novel analgesics with similar mechanisms of action.

## Author contributions

F. K., M. B., I.S., M.M.D., D.K. and D.D.P. acquisition and analysis of data, contribution to interpretation, revision and approval of final version. H. K. design of the study, contribution to writing, revision and approval of final version. S. B. conception and design of the study, contribution to interpretation, contribution to writing, revision and approval of final version. X. K. design of the study, contribution to interpretation, contribution to writing, revision and approval of final version.

## Conflict of interest

None.
